# Integrative analysis of the hypothalamic-pituitary-testicular axis reveals molecular mechanisms underlying sperm motility differences in Landes ganders

**DOI:** 10.3389/fvets.2026.1809258

**Published:** 2026-04-22

**Authors:** Yanyan Liu, Fuqiang Chang, Biyu Zhang, Haidong Liu, Qian Shi, Haitao Diao, Meng Zhou, Shouqian Sang, Xiu Li, Qianqian Hu, Jing Li, Chongmei Ruan

**Affiliations:** 1 College of Animal Science, Anhui Science and Technology University, Chuzhou, China; 2 Anhui Province Key Laboratory of Animal Nutrition Regulation and Health, Chuzhou, China; 3 Anhui Engineering Research Center of Pork Quality Control and Enhancement, Chuzhou, China

**Keywords:** HPT axis, Landes goose, sperm motility, steroidogenesis, testicular histology

## Abstract

This study aims to investigate the differences in semen quality between the high- and low-sperm motility group in Landes ganders, and to elucidate the regulatory role of the hypothalamic-pituitary-testicular (HPT) axis. Based on sperm motility values, the Landes ganders were divided into the high-sperm motility group (HSM, *n* = 3, 51.76 ± 2.53) and the low-sperm motility group (LSM, *n* = 3, 39.39 ± 2.52). Semen quality parameters, serum antioxidant indices, testosterone concentrations, and testicular histomorphology were assessed. Transcriptome sequencing (RNA-seq) was conducted on hypothalamic, pituitary, and testicular tissues to identify differentially expressed genes (DEGs), followed by Gene Ontology (GO) and KEGG pathway enrichment analyses. Sperm concentration and motility were significantly higher in the HSM group compared with the LSM group (*p* < 0.01). Ganders with low-sperm motility group exhibited increased serum malondialdehyde levels and decreased total antioxidant capacity and superoxide dismutase activity (*p* < 0.01), along with reduced testosterone concentrations (*p* < 0.05). Histological examination revealed that HSM ganders displayed well-developed seminiferous tubules with larger diameters, increased germ cell layers, and abundant mature spermatozoa, whereas LSM ganders showed impaired spermatogenesis and reduced tubular development (*p* < 0.01). Key genes associated with spermatogenesis (*SPATA1*, *WT1*) and steroidogenesis (*3β-HSD*, *CYP11A1*, *CYP19A1*) were significantly upregulated in the HSM group. Transcriptomic profiling identified 1,189, 2,126, and 1,538 DEGs in the hypothalamus, pituitary, and testis, respectively. These DEGs were significantly enriched in pathways related to GnRH signaling, steroid hormone biosynthesis, neuroactive ligand-receptor interaction, and Wnt signaling. Differences in sperm motility among Landes ganders are closely associated with antioxidant status, endocrine regulation, testicular development, and coordinated gene expression along the HPT axis. These findings provide mechanistic insights into spermatogenesis and offer a theoretical basis for improving reproductive performance in geese.

## 1 Introduction

The Landes goose represents a significant economic waterfowl species globally, primarily esteemed for its pronounced tendency toward hepatic steatosis and substantial foie gras production (1). Although reproductive performance in geese has been widely studied, research has predominantly focused on females, with comparatively less attention given to the role of males. Indeed, semen quality serves as a critical determinant of fertilization success in geese. Impaired male reproductive performance, particularly a decline in semen quality, exerts a direct and adverse impact on fertilization rates, an effect that is especially pronounced in artificial insemination systems (2). Thus, the low reproductive efficiency of ganders, notably the suboptimal fertilization rate of hatching eggs (3), has emerged as a major bottleneck constraining the sustainable development of the Landes goose industry. Relative to chickens and ducks, ganders typically demonstrate inferior reproductive performance, largely attributable to their seasonal breeding patterns and strong pair-bonding behavior. Sperm motility is a key parameter for evaluating male fertility. The testis, as the site of spermatogenesis, directly determines sperm quantity, quality, and motility through its developmental status, histological architecture, and intrinsic molecular regulatory networks. Given the reproductive limitations observed in Landes geese, identifying the major factors that determine semen quality is essential for improving reproductive efficiency and strengthening industry productivity. Semen quality directly influences the success of artificial insemination and overall reproductive outcomes in male geese. A decline in semen quality leads to reduced fertility, whereas improvements in semen traits can significantly enhance reproductive performance (4). Previous studies in waterfowl have shown that high-motility drakes exhibit greater ejaculate volume and higher sperm viability, accompanied by more developed seminiferous tubules containing tightly arranged germ cells and abundant mature spermatozoa (5). However, the sperm plasma membrane is rich in polyunsaturated fatty acids and contains relatively low levels of antioxidant enzymes in the cytoplasm, rendering them particularly vulnerable to oxidative damage. Excessive reactive oxygen species can induce lipid peroxidation, compromise membrane integrity, and impair mitochondrial function in sperm, leading to declines in motility, morphological abnormalities, and DNA damage (6). This series of processes is primarily regulated by the reproductive endocrine system, in particular via hormones secreted by the hypothalamic-pituitary-testicular (HPT) axis. The studies in Yangzhou geese have confirmed that serum testosterone levels are significantly positively correlated with testicular weight and spermatogenic function (7). However, most existing studies have examined sperm motility as a single phenotype and focused on differential gene expression within a single tissue, whereas integrative analyses across multiple reproductive regulatory organs are still lacking. As the central endocrine pathway governing male reproductive function, the HPT axis regulates testicular steroidogenesis and spermatogenesis through the secretion of GnRH from the hypothalamus, followed by LH and FSH release from the pituitary. In poultry, elucidating the molecular regulatory mechanisms of the HPT axis is essential for understanding spermatogenic efficiency, testicular development, and variation in gonadal function. Transcriptional regulation serves as the foundation of spermatogenesis, whereas RNA-seq has emerged as the method of choice for comprehensively and quantitatively profiling tissue-specific transcriptomes. The spermatogenic function of the testis is orchestrated through the coordinated action of non-coding RNAs, protein-coding genes, and various signaling pathways (8). The spermatogenic function of the testis is orchestrated through the coordinated action of non-coding RNAs, protein-coding genes, and multiple signaling pathways. Sperm motility is shaped by multiple factors, mainly involving the process of spermatogenesis in the testis and the subsequent physiological maturation of spermatozoa within the male reproductive tract—especially the ductus deferens in avian species (9). Therefore, in this study, we comprehensively evaluated semen quality, testicular developmental characteristics, histological structure, and serum antioxidant capacity in Landes ganders. By integrating transcriptomic analyses of the hypothalamus, pituitary, and testis, we aimed to identify signaling pathways and key genes associated with sperm motility, thereby providing theoretical insights into reproductive regulation and improving the selection efficiency of high-fertility male geese.

## 2 Materials and methods

### 2.1 Ethics statement

All procedures performed in this study were approved by the Institutional Animal Care and Use Committee (IACUC) of Anhui Science and Technology University (Approval No. AHSTU2025006) and were conducted in accordance with the approved guidelines.

### 2.2 Experimental design and semen quality evaluation

Prior to the formal experiment, 20 healthy 2-year-old Landes ganders with similar body weights and body conditions were selected for this study, and all birds underwent a 3-week semen collection training period. Semen was collected twice per week using the dorso-abdominal massage method (10). Semen quality was assessed within 30 min of collection. Sperm motility was evaluated using a computer-assisted sperm analysis (CASA) system (ML-608JZ, Nanning Songjing Tianlun Bioengineering Co., Ltd., Nanning, China) according to the settings described by Hao et al. (11). Based on sperm motility, the samples were divided into high-motility group (HSM, *n* = 3) and low-motility group (LSM, *n* = 3). The difference in sperm motility between the two groups was highly significant (*p* < 0.01). Semen quality parameters included semen volume, sperm concentration, semen pH, sperm motility, and sperm morphological abnormality rate. All parameters were assessed according to the methods described by Wu et al. (12).

### 2.3 Morphometric assessment and sample collection

After a 12-h fasting period, the body weight of each gander was recorded. Bilateral testes and the vas deferens were then excised. Testicular weights (left, right, and combined) were measured using an electronic balance. The length of the vas deferens and the length, width, and depth of the testes were measured using a vernier caliper, following the morphological assessment methods described by Yan et al. (13). Blood samples were collected from the brachial vein into serum tubes without anticoagulant, allowed to clot at room temperature for 30 min, and then centrifuged at 3500×g for 10 min at 4 °C. The serum was stored at −80 °C for subsequent analysis of antioxidant capacity and reproductive hormones. Testicular tissues were fixed in 4% paraformaldehyde for histological paraffin sectioning. Hypothalamus, pituitary, and testicular tissue samples were preserved in RNA stabilization solution and subsequently stored at −80 °C for transcriptome sequencing and gene expression analysis.

### 2.4 Measurement of MDA content and T-AOC, CAT, and SOD activities

The levels of malondialdehyde (MDA; Nanjing Jiancheng, A003-1, China), total antioxidant capacity (T-AOC; Nanjing Jiancheng, A015-2-1, China), catalase (CAT; Nanjing Jiancheng, A007-1-1, China), and superoxide dismutase (SOD; Nanjing Jiancheng, A001-3, China) were determined strictly according to the instructions provided with the commercial kits. Absorbance was measured using a microplate reader (Multiskan FC1510, Thermo Scientific, United States), and the final values were calculated following the formulas supplied in the manuals. Each sample was analyzed in six technical replicates.

### 2.5 Determination of testosterone levels

Serum testosterone levels were measured using a competitive enzyme-linked immunosorbent assay (ELISA) (ARE06-JZ Testosterone ELISA Kit, Hudbio, China). The specific procedures were performed according to the method described by El Tahir et al. (14). The assay was performed strictly following the manufacturer’s instructions. Absorbance was read at 450 nm using a microplate reader.

### 2.6 Histological evaluation of testicular tissue

Testicular tissue samples were cut into small pieces of approximately 2 mm^3^ and fixed in 4% paraformaldehyde for 24 h at room temperature. After fixation, the tissues were trimmed in a fume hood and placed into dehydration cassettes. Gradient dehydration and paraffin embedding were performed sequentially using ethanol solutions of increasing concentrations, followed by alcohol-xylene, xylene, and paraffin. The embedded tissues were then sectioned at a thickness of 4-5 μm using a microtome. Hematoxylin and eosin (H&E) staining was conducted following the protocol described by Song et al. (15). The stained sections were examined and imaged using a fully automated digital slide scanner (BA600-4, MOTIC, China). Cell quantification within the testicular tissue was performed using Image-Pro Plus 6.0 software.

### 2.7 RNA extraction and sequencing

Approximately 20 mg of tissue was homogenized in lysis buffer, and total RNA was extracted using TRNzol Universal Reagent (Tiangen Biotech, Beijing, China) according to the manufacturer’s instructions. RNA concentration and purity were measured using a NanoDrop One spectrophotometer (Thermo Scientific, United States), RNA integrity was assessed using an Agilent 2100 Bioanalyzer, and samples with RNA integrity number (RIN) values between 8 and 10 were used for library construction. The extracted RNA samples were stored at −80 °C until further use. Sequencing libraries were prepared with the Illumina Stranded mRNA Prep kit (Illumina, San Diego, United States) and sequenced on the Illumina NovaSeq X Plus platform using a 2 × 150 bp paired-end strategy.

### 2.8 Transcriptome alignment and assembly

Raw sequencing reads were filtered using Fastp (version 0.23.1) to obtain high-quality clean reads. The clean reads were then aligned to the goose reference genome using HISAT (16). The resulting SAM files were converted to BAM format and sorted using SAMtools.

### 2.9 Identification and functional analysis of differentially expressed genes

Differentially expressed genes (DEGs) between groups were identified using DESeq2 (17). Significant differential expression was identified based on the criteria |Log2(Foldchange)| ≥ 1 and *p* < 0.05. Functional enrichment analyses of the DEGs, including Gene Ontology (GO) and Kyoto Encyclopedia of Genes and Genomes (KEGG) pathways, were performed using online analysis platforms, with the gray goose (*Anser cygnoides*) set as the reference species.

### 2.10 Primer design and validation

Primers were designed using Primer 5.0 (Supplementary Table 1), and synthesized by Shanghai Sangon Bioengineering Co., Ltd. GAPDH was used as the internal control. Seven spermatogenesis-related genes and nine randomly selected DEGs were validated. Complementary DNA (cDNA) was synthesized from the extracted RNA using the 5 × Evo M-MLV RT Reaction Mix Ver.2 kit (Accurate Biology, AG11706, China) and stored at −20 °C. qRT-PCR was performed using SYBR Green SupTaq HS (Accurate Biology, AG11761, China) on a fluorescence-based qPCR system (BIO-96, Bioer Technology, China) with a total reaction volume of 10 μL. Cycling conditions were: 94 °C for 5 min, followed by 40 cycles of 94 °C for 10 s, 60 °C for 20 s, and 72 °C for 20 s. Melting curve analysis was conducted at 60 °C for 25 s. Relative gene expression was calculated using the 2^−ΔΔCt^ (18)method, with six technical replicates per sample.

### 2.11 Data analysis

Data were analyzed using SPSS 26.0 (IBM, Armonk, NY, United States). Using independent samples *t*-tests, and the results are expressed as mean ± standard deviation (SD). *p* < 0.05 was considered statistically significant, *p* < 0.01 as highly significant, and *p* > 0.05 as not significant. Relative gene expression levels were calculated using the 2^−ΔΔCt^ method, and the results are presented in figures generated with GraphPad Prism 9 (La Jolla, CA, United States).

## 3 Results

### 3.1 Differences in semen quality between HSM and LSM of Landes geese

**Figure 1:**
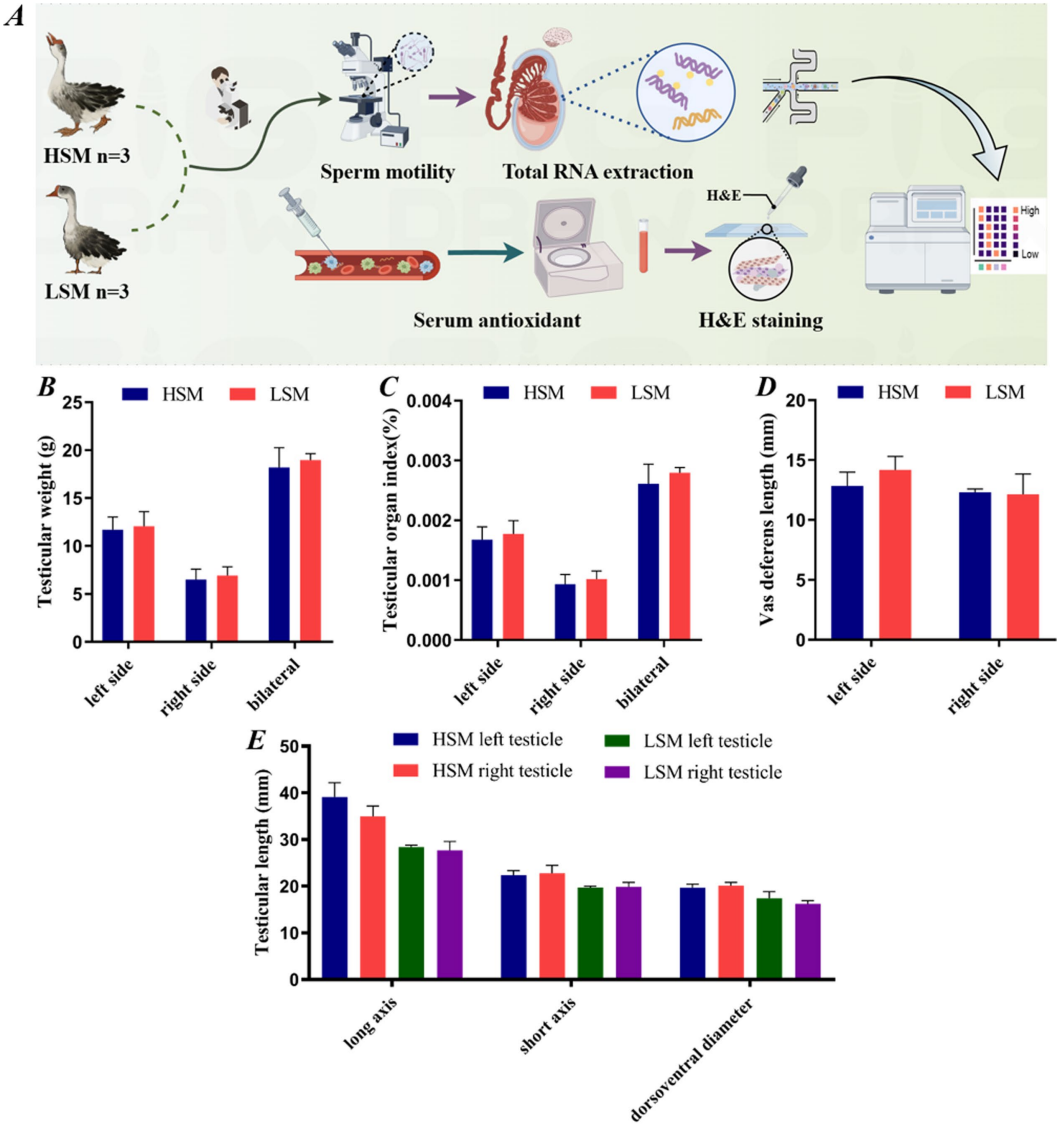
Morphological analysis of testes in high- and low-sperm motility geese. **(A)** Experimental flowchart; **(B)** Testis weight; **(C)** Testis organ index; **(D)** Vas deferens length; **(E)** Testis morphological parameters. “*”*p* < 0.05, “**”*p* < 0.01; the same applies to the following figures.

According to sperm motility, the Landes geese semen was divided into two groups: the high-sperm motility (HSM) group and the low-sperm motility (LSM) group (Figure 1A). As shown in Table 1, the sperm motility and sperm density of the HSM group were significantly higher than those of the LSM group (*p* < 0.01). There were no significant differences in semen volume, pH, and sperm deformity rate between the HSM and LSM groups (*p* > 0.05).

**Table 1:** Differences in semen quality of Landes geese between high- and low-sperm motility groups.

Item	HSM	LSM	*p*-value
Semen volume (mL)	0.28 ± 0.02	0.29 ± 0.03	0.322
pH	7.30 ± 0.16	7.31 ± 0.16	0.795
Sperm concentration (10^8^/mL)	1.60 ± 0.05^A^	0.84 ± 0.14^B^	0.000
Sperm motility (%)	51.76 ± 2.53^A^	39.39 ± 2.52^B^	0.000
Sperm deformity (%)	11.66 ± 2.32	11.41 ± 2.09	0.635

Values within a row with different superscript letters are significantly different (*p* < 0.01).

### 3.2 Morphological analysis of testes in HSM and LSM of Landes geese

As shown in Figure 1, there were no significant differences in the left testis, right testis, or both testes weight between the HSM and LSM groups (*p* > 0.05). The testicular organ index, vas deferens length, and testis length were also not significantly different between the HSM and LSM groups (*p* > 0.05). Although the testis length was higher in both the unilateral and bilateral testes of the HSM group compared to the LSM group, the difference was not significant (*p* > 0.05).

### 3.3 Comparative analysis of serum MDA, T-AOC, SOD, and CAT in HSM and LSM of Landes geese

**Figure 2:**
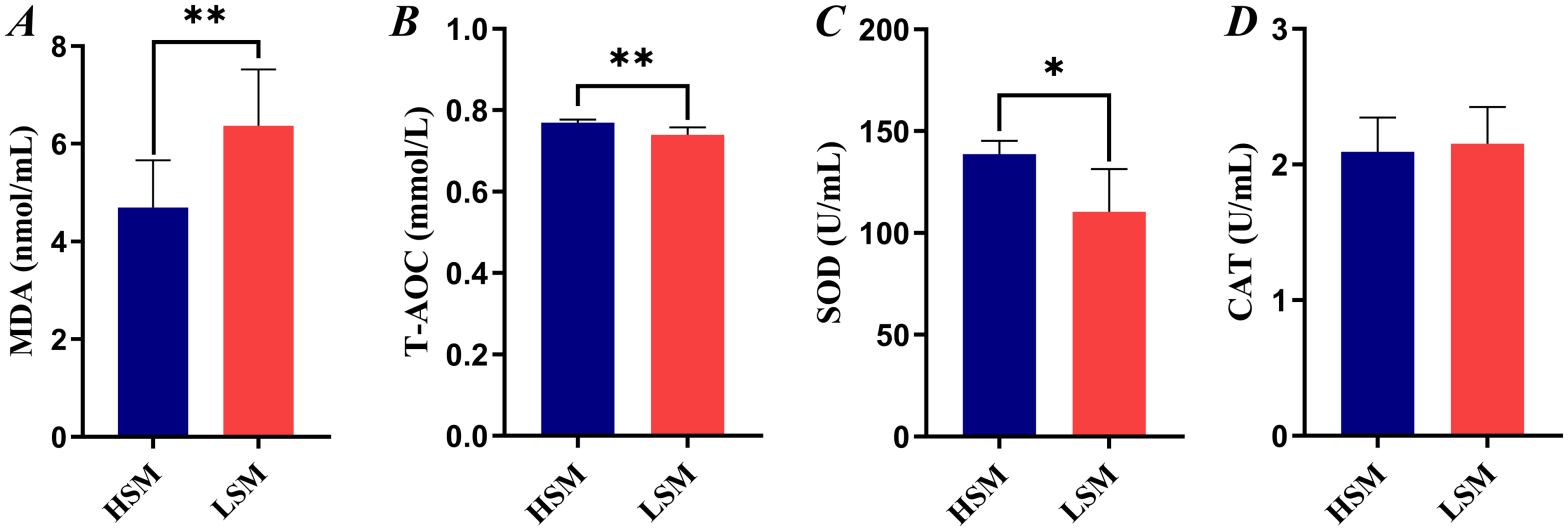
Serum oxidative stress and antioxidant indices in HSM and LSM groups of Landes ganders. **(A)** Malondialdehyde (MDA) levels; **(B)** total antioxidant capacity (T-AOC); **(C)** superoxide dismutase (SOD) activity; **(D)** catalase (CAT) activity.

As shown in Figure 2, the MDA content in the LSM group was higher than that in the HSM group (*p* < 0.01). The SOD levels in the HSM group were higher than those in the LSM group (*p* < 0.05), while the CAT content in the LSM group was higher than that in the HSM group (*p* > 0.05).

### 3.4 Comparative analysis of testosterone levels in HSM and LSM of Landes geese

**Figure 3:**
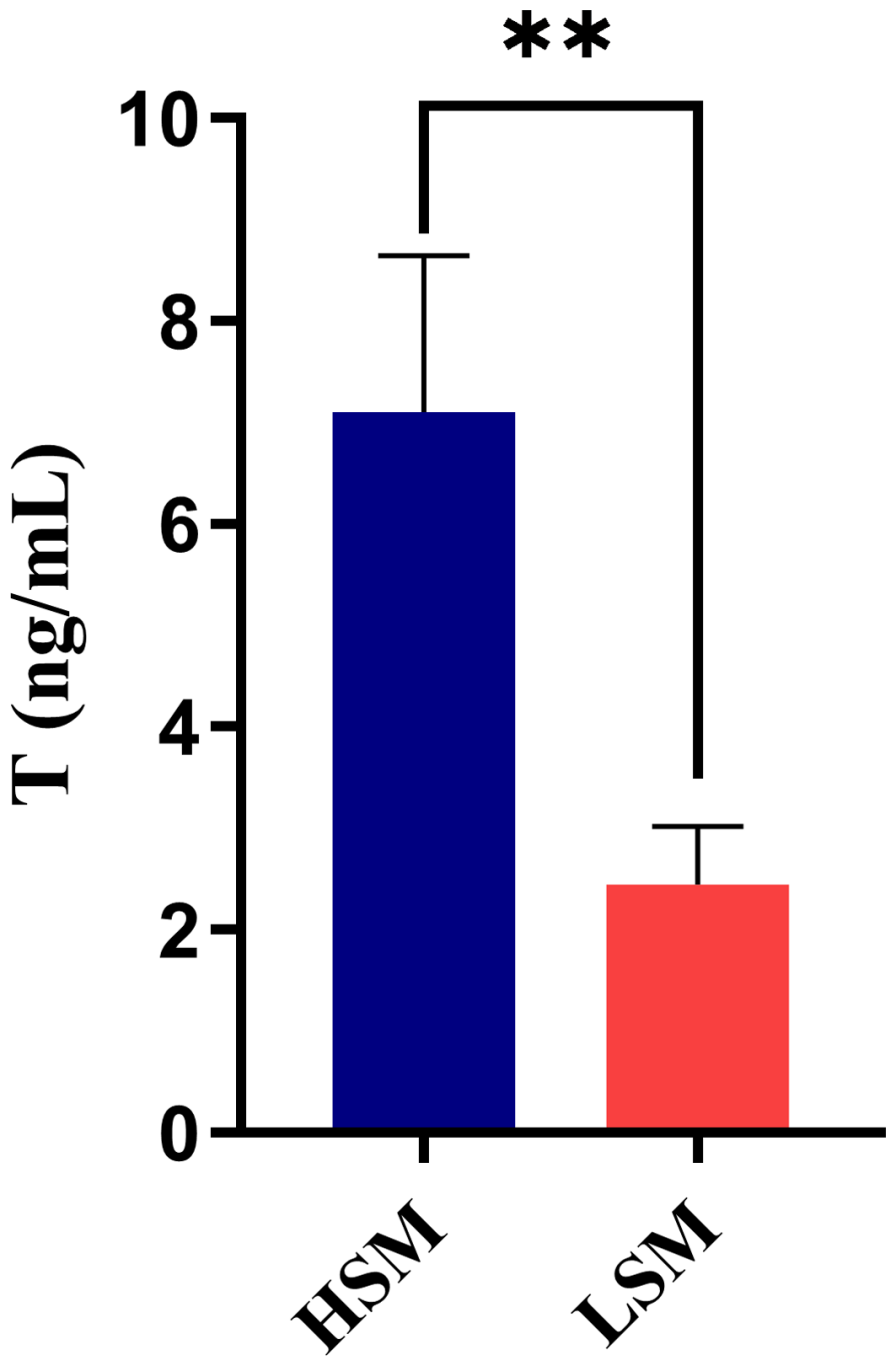
Comparative analysis of testosterone levels in HSM and LSM of Landes geese.

Blood samples were collected from geese in HSM and LSM to determine the testosterone levels. As shown in Figure 3, the testosterone level in the HSM group was higher than that in the LSM group (*p* < 0.01).

### 3.5 Comparative histological analysis of testes in HSM and LSM of Landes geese

**Figure 4:**
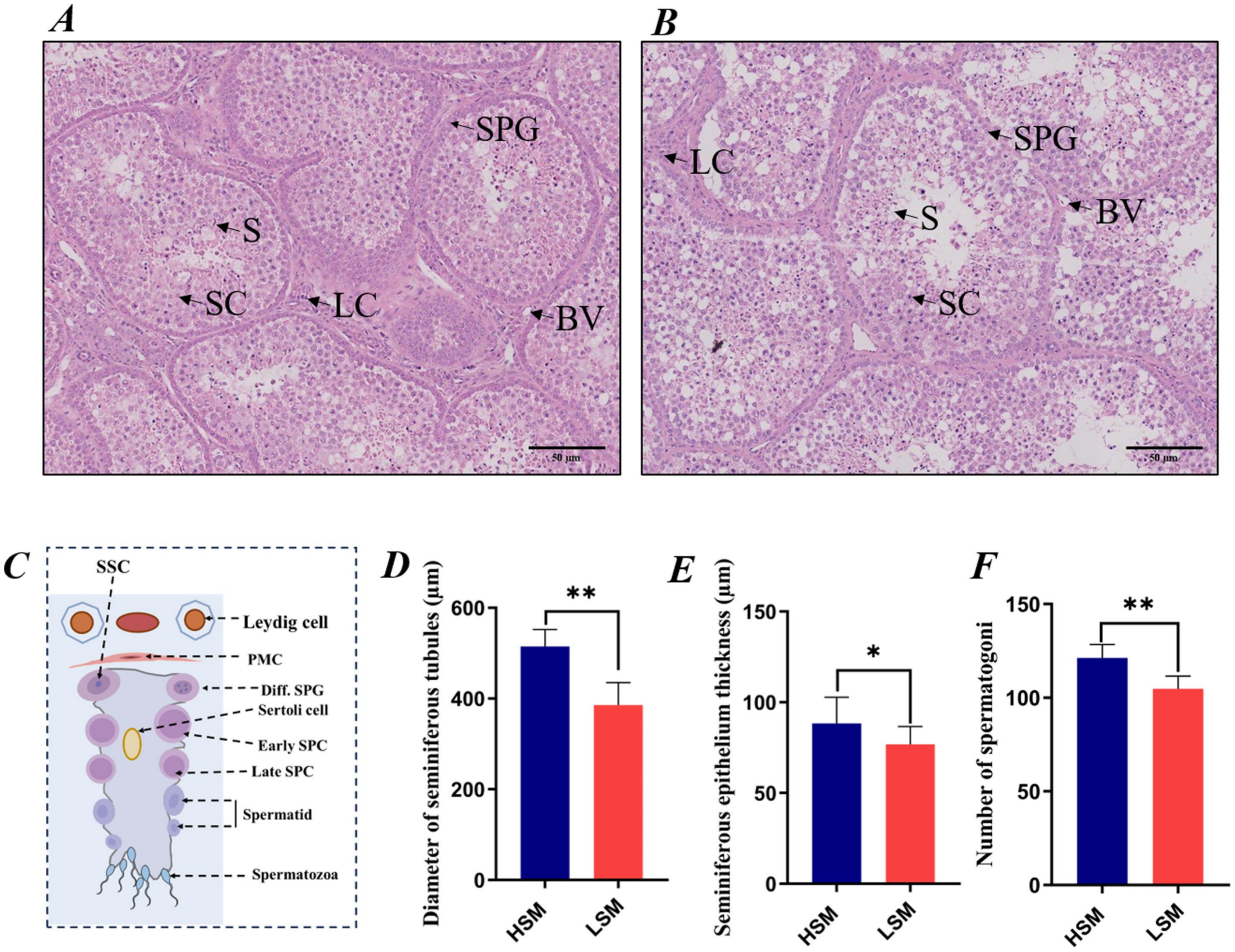
Comparative histomorphological analysis of testes in HSM and LSM of Landes geese: **(A)** Testicular histological structure in the HSM group; **(B)** Testicular histological structure in the LSM group; **(C)** Schematic diagram of a seminiferous tubule; **(D)** Diameter of seminiferous tubules; **(E)** Thickness of the spermatogenic epithelium; **(F)** Number of spermatogonia. LC, Leydig cell; SPG, Spermatogonium; S, Spermatozoon; BV, Blood vessel; SC, Sertoli cell.

Histological changes in the testes were assessed using hematoxylin and eosin (H&E) staining. In the HSM group, the basement membrane of the seminiferous tubules appeared distinct, with spermatogenic epithelial cells exhibiting a compact arrangement, and interstitial cells displaying normal morphology. In contrast, in the LSM group, there was a progressive widening of the gaps between the seminiferous tubules, a reduction in the number of interstitial cells, and a blurring of the seminiferous tubule wall structure. As shown in Figure 4, the diameter of the seminiferous tubules and the number of spermatogonia in the HSM group were significantly higher than those in the LSM group (*p* < 0.01), while the thickness of the spermatogenic epithelium was also higher in the HSM group than in the LSM group (*p* < 0.05).

### 3.6 The mRNA expression levels of genes related to spermatogenesis in testes in HSM and LSM of Landes geese

**Figure 5:**
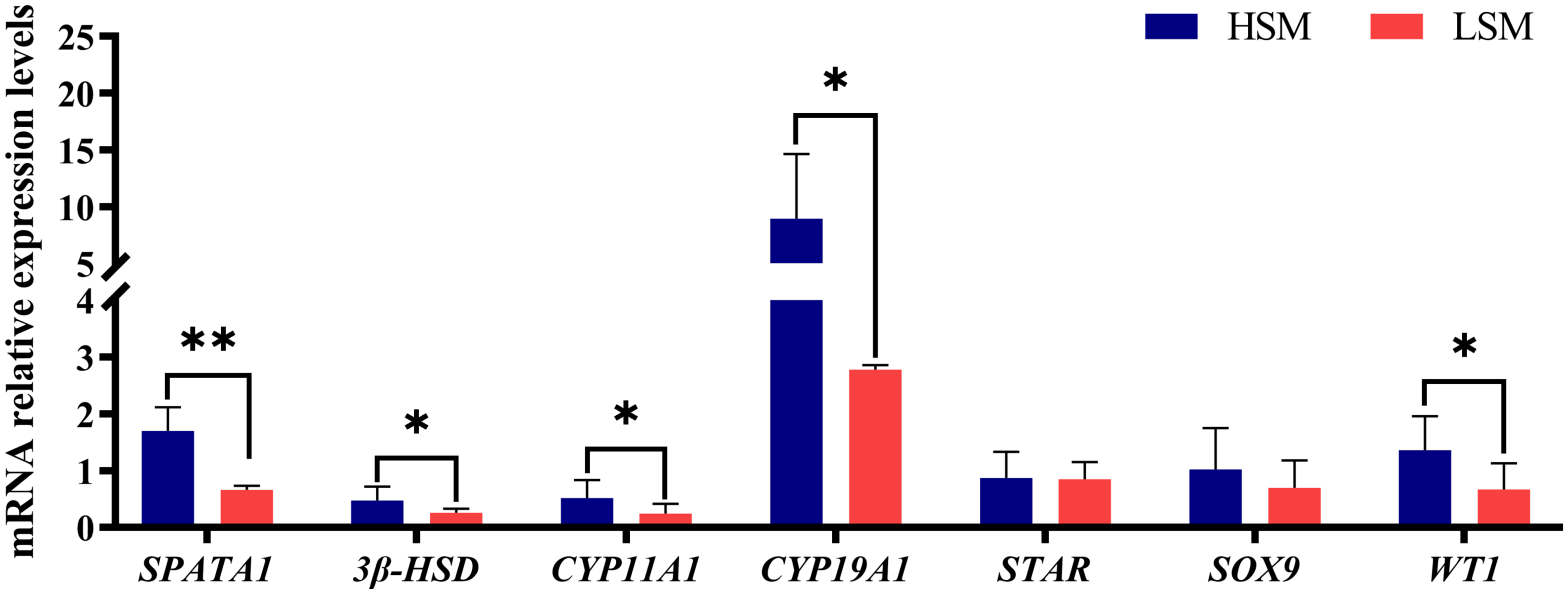
The mRNA expression levels of genes related to spermatogenesis in testes in HSM and LSM of Landes geese.

As shown in Figure 5, the expression levels of *SPATA1*, *3β-HSD*, *CYP11A1*, *CYP19A1*, *STAR*, *SOX9*, and *WT1* were all elevated in the HSM group compared with the LSM group. Among these, S*PATA1* expression was significantly higher in the HSM group than in the LSM group (*p* < 0.01). The expression levels of *3β-HSD*, *CYP11A1*, *CYP19A1*, and *WT1* were significantly higher in the HSM group than in the LSM group (*p* < 0.05), while *STAR* and *SOX9* showed no significant differences between the two groups.

### 3.7 RNA-seq identification of differentially expressed genes

**Figure 6:**
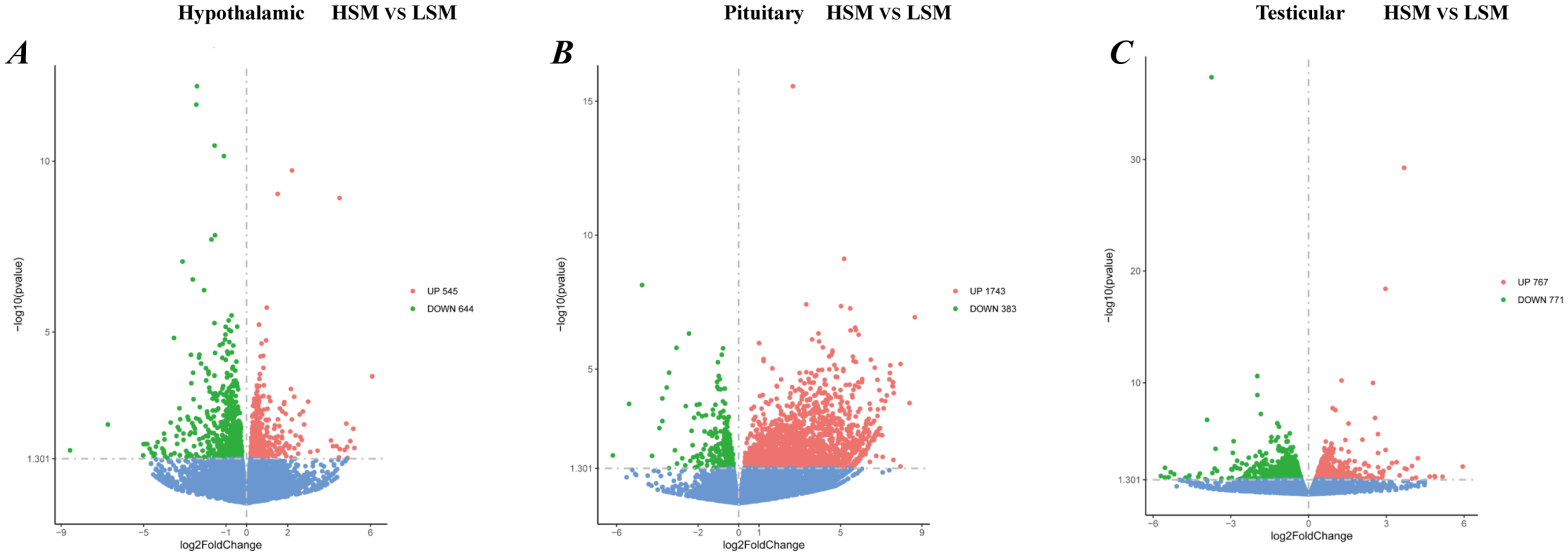
Identification of differentially expressed genes in the hypothalamus, pituitary, and testis of Landes geese with HSM and LSM. **(A–C)** Volcano plots showing DEGs between HSM and LSM in the hypothalamus, pituitary, and testis, respectively.

To investigate the molecular mechanisms underlying sperm motility and HPT axis development in Landes geese, transcriptome sequencing was performed on hypothalamus, pituitary, and testis tissues from HSM and LSM groups. As summarized in Supplementary Table 2, a total of 841,803,566 raw reads were obtained from 18 samples, and after stringent quality control, an average of 45,142,009 high-quality clean reads per sample were retained. Supplementary Figure 1 PCA demonstrated that samples clustered clearly according to experimental groups, indicating high sequencing quality and good biological reproducibility, suitable for downstream analyses. Differential expression analysis identified 1,189 DEGs in the hypothalamus (545 upregulated and 644 downregulated), 2,126 DEGs in the pituitary (1743 upregulated and 383 downregulated), and 1,538 DEGs in the testis (767 upregulated and 771 downregulated) between HSM and LSM (Figure 6).

### 3.8 Differentially expressed genes functional analysis

**Figure 7:**
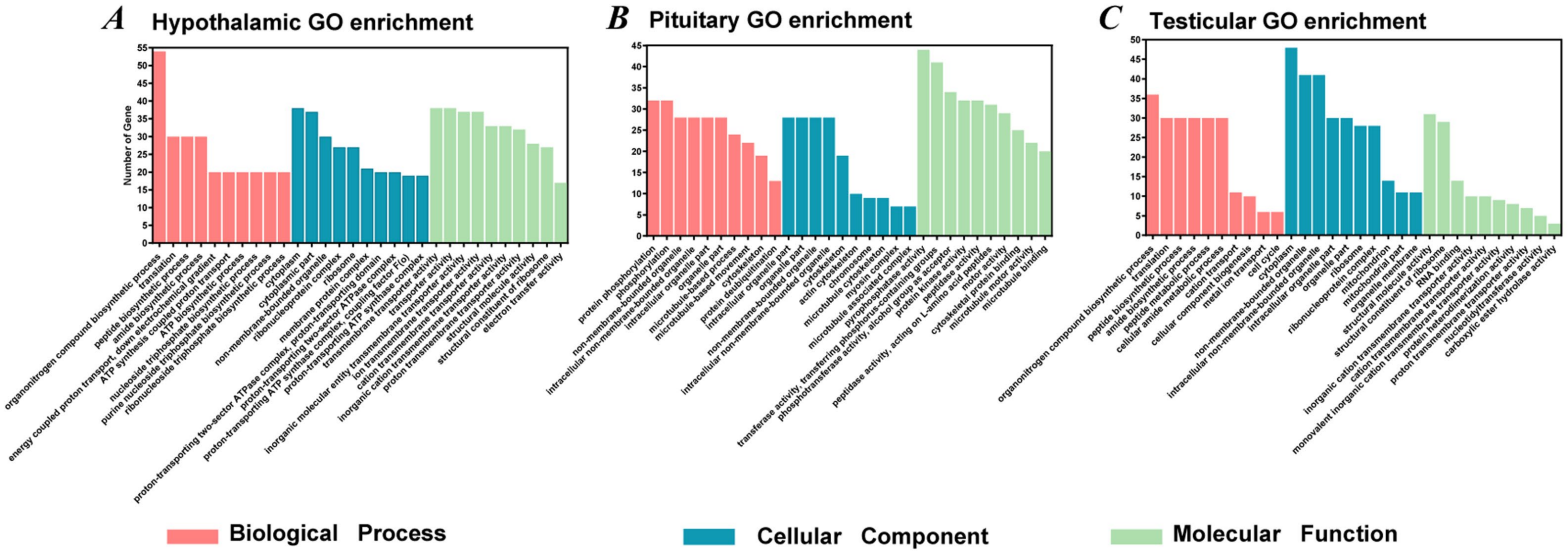
GO enrichment analysis of the hypothalamus, pituitary, and testis in Landes geese with high (HSM) and low (LSM) sperm motility. Panels **(A–C)** represent GO enrichment analyses of the hypothalamus, pituitary, and testis, respectively, between the HSM and LSM groups.

Gene Ontology (GO) enrichment analysis was performed to investigate the potential biological functions of the differentially expressed genes (DEGs) identified between the HSM and LSM groups in the hypothalamus, pituitary, and testis. As shown in Figure 7, the enriched GO terms revealed several key biological processes associated with cellular metabolism, protein synthesis, and signal transduction. In the hypothalamus, the DEGs were predominantly enriched in biological processes related to protein biosynthesis and cellular energy metabolism, including translation, peptide biosynthetic processes, ATP biosynthetic processes, and nucleoside triphosphate biosynthetic processes. These enrichments suggest enhanced metabolic activity and protein synthesis in hypothalamic cells, which may support the high energy demand associated with neuroendocrine regulation of reproductive functions. In the pituitary, enriched GO terms were mainly associated with protein phosphorylation, cytoskeletal organization, and intracellular signaling processes. These processes are closely related to hormone synthesis, secretion, and intracellular signal transduction in endocrine cells, indicating that changes in pituitary cellular signaling and structural dynamics may contribute to differences in reproductive regulation between the two groups. In the testis, the DEGs were primarily enriched in processes related to protein synthesis, cellular metabolism, and cell cycle regulation. These functional categories are closely associated with the proliferation and differentiation of spermatogenic cells, suggesting that transcriptional regulation of these processes may influence spermatogenesis and ultimately affect sperm motility.

### 3.9 KEGG pathway enrichment analysis

**Figure 8:**
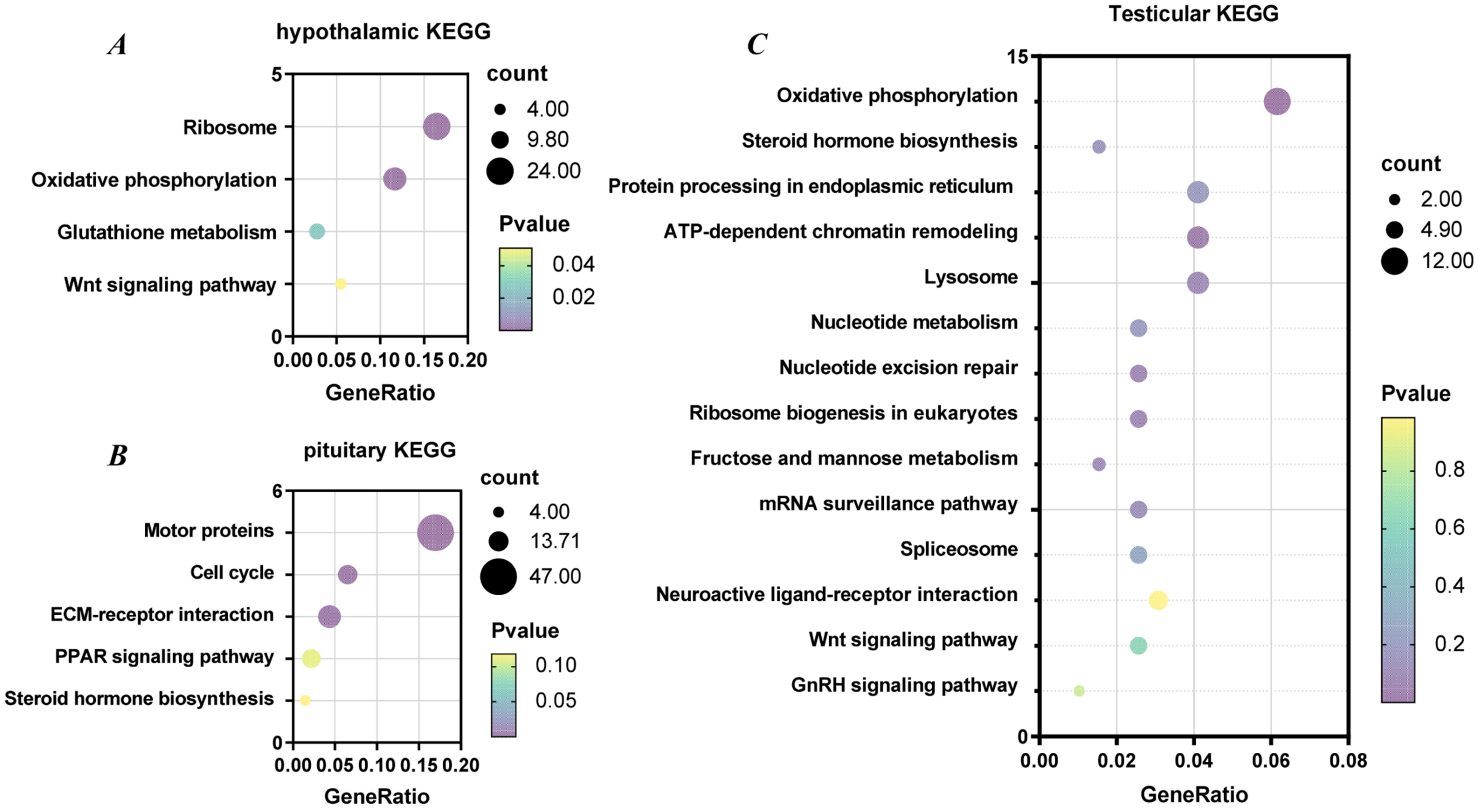
Significantly enriched KEGG pathways in the **(A)** hypothalamus, **(B)** pituitary, **(C)** testis.

KEGG enrichment analysis identified 10, 4, and 14 significantly enriched pathways in the hypothalamus, pituitary, and testis, respectively. In the hypothalamus, the most enriched pathways were Ribosome, Oxidative phosphorylation, Glutathione metabolism, and Wnt signaling pathway. The pituitary was mainly enriched in pathways associated with Motor proteins, Cell cycle regulation, ECM–receptor interaction, the PPAR signaling pathway, and Steroid hormone biosynthesis. In the testis, the enriched pathways were predominantly related to the GnRH signaling pathway, Steroid hormone biosynthesis, Wnt signaling pathway, ATP-dependent chromatin remodeling, Neuroactive ligand-receptor interaction and Lysosome function (Figure 8). These findings suggest that multiple pathways related to energy metabolism, endocrine regulation, and reproductive signaling may collectively contribute to the regulation of sperm motility in Landes geese.

### 3.10 Validation of RNA-seq

**Figure 9:**
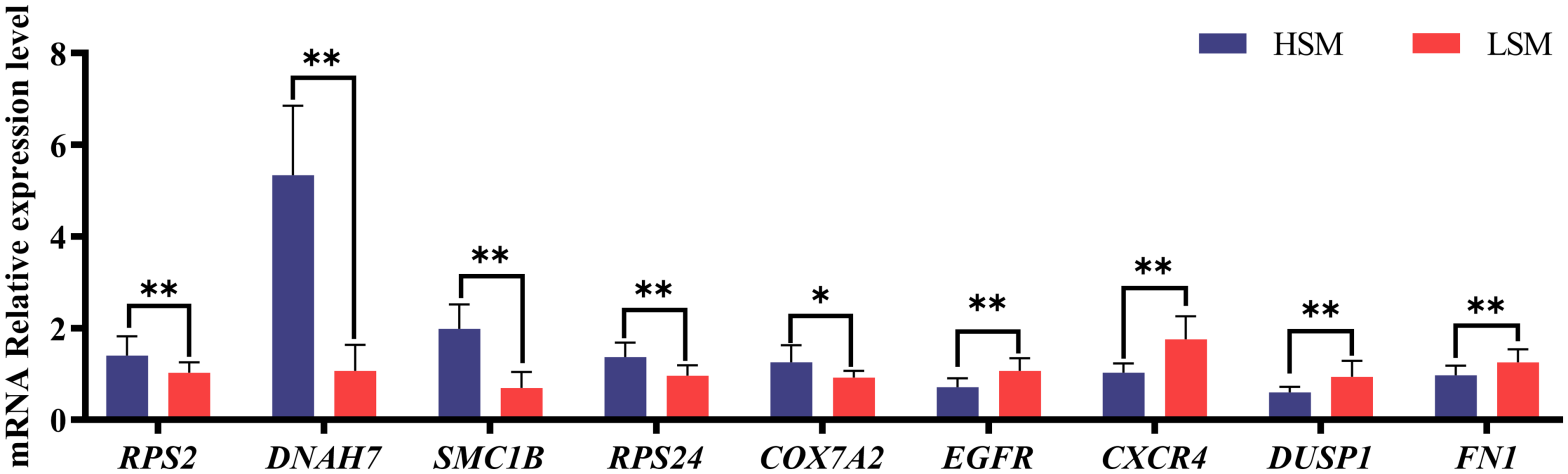
Validation of RNA-seq results by qRT-PCR. Total RNA was extracted from hypothalamus, pituitary, and testis tissues and analyzed by qRT-PCR.

To verify the reliability of the RNA-seq data, nine DEGs were selected for qRT-PCR analysis, with *GAPDH* as the internal reference gene. Among them, *RPS2*, *DNAH7*, *SMC1B*, *RPS24*, and *COX7A2* were significantly upregulated in the HSM group compared with the LSM group, whereas *EGFR*, *CXCR4*, *DUSP1*, and *FN1* were downregulated. The expression patterns detected by qRT-PCR were consistent with the RNA-seq results, confirming the reliability of the sequencing data (Figure 9).

## 4 Discussion

Semen quality is a key parameter for assessing the reproductive capacity of male poultry. In the present study, highly significant differences in both sperm motility and concentration were observed between the high- and low-sperm motility groups of Landes geese, further highlighting variations in individual reproductive potential. Although the semen volume of Landes geese in this study was consistent with values reported for other breeds, such as the White Kołuda (19) and several Polish goose breeds (20), semen volume alone is an unreliable predictor of reproductive efficiency. Instead, key determinants of male fertility, such as sperm motility, viability, and concentration, should be considered in an integrated assessment.

Physiological parameters of animals provide important information regarding their growth and overall functional status. The testis serves as the site of spermatogenesis as well as androgen secretion. Morphological features, weight, and histological characteristics of the testis vary significantly across developmental stages. These variations can directly influence spermatogenic function. This study used Landes geese as the experimental model. Morphological comparisons of the testes and vas deferens were conducted between the high-sperm motility (HSM) and low-sperm motility (LSM) groups. The results indicated no significant differences in testis weight or vas deferens length between the two groups. However, testis length was significantly greater in the HSM group compared to the LSM group. Li et al. (21) previously reported that testis weight, gonadosomatic index (GSI), and histological parameters were significantly lower in azoospermic ganders than in the normal spermatogenesis group, which may be associated with structural and functional abnormalities in the testis. In male birds, ejaculation frequency, ejaculate volume, and sperm concentration have been reported to increase with greater testicular mass (22). Thus, changes in the testicular index reflect alterations in spermatogenic function, with a reduced index reflecting diminished spermatogenic capacity.

The present study revealed a significant difference in the degree of oxidative stress between the high- and low-sperm motility groups. Specifically, the HSM group exhibited significantly lower levels of malondialdehyde, a terminal product of lipid peroxidation, along with significantly higher activities of the antioxidant enzyme superoxide dismutase and total antioxidant capacity. This profile indicates a more favorable redox status in the high-motility sperm. These findings suggest that the lower MDA levels correspond to reduced peroxidative damage to the sperm plasma membrane, which is rich in oxidation-sensitive polyunsaturated fatty acids. Maintaining membrane integrity is critical for preserving fluidity, facilitating the acrosome reaction, and ultimately supporting fertilizing capacity. In fact, enhancing total antioxidant capacity through dietary supplementation with antioxidants such as vitamin E and selenium has been shown to improve sperm function (23). Concurrently, the superior antioxidant defense likely more effectively neutralizes excessive reactive oxygen species, thereby protecting sperm DNA integrity and mitochondrial function from oxidative damage (24). This is particularly crucial, as oxidative stress can induce DNA damage and apoptosis mediated by genes such as Bax and Caspase-3, directly leading to loss of sperm motility and reduced fertility (25).

Testosterone is a key biomarker of reproductive health in male geese and is closely associated with reproductive performance. During the breeding season, serum testosterone levels in ganders fluctuations significantly. There is a positive correlation between testosterone concentration and reproductive performance, with higher testosterone levels corresponding to enhanced reproductive potential (26). Testosterone not only influences sexual behavior in male geese but also plays a crucial role in the development and function of the reproductive organs. At the onset of the breeding season, increased and testosterone levels promote testicular development and spermatogenesis, thereby enhancing reproductive performance. Conversely, as reproductive performance declines, the reduced testosterone levels lead to testicular regression, impairing reproductive efficiency. High testosterone levels during the breeding season stimulate spermatogonial differentiation and spermatocyte maturation, resulting in increased sperm quantity and quality. In the non-breeding season, decreased testosterone levels decrease, accompanied by a reduction in testicular volume, seminiferous tubule diameter, and epithelial height, ultimately leading to a marked decline in spermatogenic activity (27). In the present study, testosterone levels in the high-sperm motility group were higher than those in the low-sperm motility group. The elevated testosterone level likely promotes spermatogenesis and enhances sperm motility.

A Spermatogenesis is a complex and dynamic biological process that encompasses the transition from spermatogonial stem cells to mature spermatozoa. This process relies on the continuous division and differentiation of spermatogonial stem cells and requires the coordinated expression of multiple genes in the testes. Previous studies have shown that genes expressed in the testes during the breeding season are primarily associated with developmental processes. The *SPATA1* gene plays a crucial role in spermatogenesis, particularly in the formation and functional regulation of spermatocytes. *SPATA1* is primarily localized to the acrosomal region of developing spermatids (28). Studies suggest that *SPATA1* may influence sperm morphology and fertility by regulating the structure and function of spermatids (29). *3β-HSD* is widely expressed in the testes, ovaries, and adrenal glands. In the testes, the enzymatic activity of *3β-HSD* is closely linked to the steroidogenic capacity of Leydig cells (30). As a critical rate-limiting enzyme in the biosynthesis of steroid hormones, *3β-HSD* directly affects the levels of sex hormones by catalyzing the conversion of pregnenolone to progesterone, a key step that precedes the synthesis of androgens and estrogens (31). Reduced expression of *3β-HSD* has been shown to lower testosterone and estrogen levels, thereby impairing reproductive function (32). In avian reproduction, both *CYP11A1* and *CYP19A1* are essential, as they initiate the biosynthetic pathways of sex hormones and exert direct influence on reproductive capacity. These genes operate in close coordination within the steroidogenic pathway: *CYP11A1* initiates the process by converting cholesterol into pregnenolone, while *CYP19A1* regulates the male reproductive system by aromatizing androgens into estrogens (33). *CYP19A1* also plays a key role in maintaining estrogen levels, which are essential for oocyte maturation and ovulation in females, and is expressed in Leydig cells of the testis to regulate local estrogen synthesis (34). *STAR* (Steroidogenic Acute Regulatory Protein) is a critical regulator of steroid hormone biosynthesis. It supports testosterone synthesis and maintains the testicular microenvironment during spermatogenesis (35). The transfer of cholesterol to the inner mitochondrial membrane by *STAR*, followed by *CYP11A1* catalyzed conversion to pregnenolone, represents the core step of steroid hormone synthesis. Mutations in *STAR* result in disorganized testicular architecture, seminiferous tubule obstruction, reduced semen volume, and increased sperm abnormalities, ultimately leading to reduced fertility (36). Additionally, *STAR* mutations are associated with lipid accumulation, upregulation of steroidogenic enzymes, and significant declines in androgen levels. *SOX9* indirectly influences sperm production and maturation by regulating Sertoli cell function. The expression level of *SOX9* is closely associated with the proliferation and differentiation of Sertoli cells, whose normal function is critical for maintaining a stable microenvironment for spermatogenesis (37). Furthermore, *SOX9* plays a key role in sex determination and testicular development by regulating the transcriptional activity of genes involved in gonadal development through chromatin domains. Aberrant *SOX9* expression in poultry may lead to reproductive disorders or even infertility (38). *WT1* maintains Sertoli cell polarity by modulating the Wnt signaling pathway, and is essential for the formation and function of the blood-testis barrier (BTB). The BTB is a critical structure that protects germ cells from harmful substances during spermatogenesis (31). Loss of *WT1* function disrupts BTB integrity, resulting in spermatogenic failure (39). In this study, the expression levels of *SPATA1*, *3β-HSD*, *CYP11A1*, *CYP19A1*, and *WT1* were significantly higher in the high-sperm motility group compared to the low-sperm motility group. These findings suggest that enhanced spermatogenic activity and steroidogenic capacity may underlie the superior sperm motility observed in the HSM group. The coordinated upregulation of genes involved in steroid hormone biosynthesis and Sertoli cell function indicates that an optimized testicular microenvironment may promote efficient spermatogenesis and improve sperm quality. In particular, increased expression of steroidogenic genes may facilitate higher androgen production, thereby supporting the maturation and functional competence of spermatozoa.

In the field of poultry breeding, semen quality is one of the core traits determining reproductive efficiency. Nevertheless, traditional breeding methods aimed at enhancing semen quality are often protracted and inefficient due to the trait’s typically low heritability. Recent advancements in molecular biology have introduced novel strategies to overcome this challenge. In particular, RNA-seq technology enables comprehensive analysis of transcriptomic profiles across different tissues, revealing gene expression patterns and facilitating the identification of candidate genes associated with semen quality. This approach offers a theoretical basis for improving semen traits in poultry breeding and enables the discovery of potential molecular markers. Previous studies have demonstrated that transcriptomic analysis of the hypothalamic-pituitary-testicular axis has made significant progress in elucidating the molecular regulatory mechanisms of male reproductive development, identifying key pathways, and screening candidate genes (40). To further investigate the molecular regulation of sperm motility, we performed RNA-seq analysis of the hypothalamus, pituitary, and testes in high- and low-sperm motility Landes geese. A total of 1,189, 2,126, and 1,538 differentially expressed genes (DEGs) were identified in the hypothalamus, pituitary, and testes, respectively. Functional enrichment analysis revealed that these DEGs were primarily associated with reproductive regulation and energy metabolism, indicating that genes in the HPT axis play crucial roles in the formation and maintenance of sperm motility. In the hypothalamus, the majority of DEGs were significantly enriched in GO terms related to translation, peptide biosynthetic process, energy-coupled proton transport, ATP synthesis coupled proton transport, ATP biosynthetic process, and nucleoside triphosphate biosynthetic process. Concurrently, KEGG pathway analysis revealed significant enrichment in ribosome, oxidative phosphorylation, glutathione metabolism, and Wnt signaling pathways. These findings collectively suggest that hypothalamic neurons experience significant metabolic and functional remodeling during critical stages of spermatogenesis. This remodeling is characterized by the establishment of a robust material and energy foundation to meet the high-energy demands associated with reproductive activity. In the pituitary, DEGs between the HSM and LSM groups were predominantly enriched in pathways related to cell cycle, motor proteins, ECM-receptor interaction, PPAR signaling, and steroid hormone biosynthesis, consistent with previous reports (41). The PPAR signaling pathway, as a key regulator of lipid metabolism and energy homeostasis, may provide essential lipid precursors and energy to support the high-intensity hormone synthesis activity of pituitary cells. The enrichment of the steroid hormone biosynthesis pathway strongly suggests the potential local synthesis of neurosteroids, such as progesterone and estrogens, within the pituitary. These locally synthesized steroids may fine-tune the functional status of gonadotrope cells via autocrine or paracrine mechanisms. The large number of DEGs detected across the three tissues further suggests that sperm motility is not regulated by a single organ but rather by the integrated activity of the entire hypothalamic-pituitary-testicular (HPT) axis. The coordinated transcriptional changes observed in these tissues highlight the systemic regulatory mechanisms underlying semen quality in geese.

In the testes, DEGs were significantly enriched in GO terms related to translation, peptide biosynthetic process, and amide biosynthetic process. KEGG pathway analysis further revealed activation of key pathways, including GnRH signaling, steroid hormone biosynthesis, Wnt signaling, ATP-dependent chromatin remodeling, neuroactive ligand–receptor interaction, and lysosome function. These findings collectively depict that during critical stages of spermatogenesis, the testes receive LH and FSH signals from the central axis (pituitary) and actively coordinate gene expression, protein synthesis, cellular remodeling, and signal transduction to precisely drive spermatogenic processes, ultimately determining semen quality. Previous studies on the goose testicular transcriptome have identified numerous DEGs involved in reproductive regulation, metabolic processes, and signal transduction. Moreover, transcriptome analyses across different reproductive stages have shown significant enrichment in neuroactive ligand–receptor interactions, Wnt signaling, GnRH signaling, and various metabolic pathways, suggesting that these key regulatory pathways may play pivotal roles in modulating sperm motility (42).

Further analysis revealed that *RPS2*, *RPS24*, *DNAH7*, *SMC1B*, and *COX7A2* were all upregulated in HSM geese, and these genes are closely associated with spermatogenesis. Previous studies have shown that *RPS2* is linked to infertility phenotypes in testicular transcriptomes, while *RPS24* is essential for sperm formation and male fertility in adult Drosophila, with its deficiency leading to abnormal chromosome segregation and cytokinesis during meiosis, impaired sperm elongation, and axonemal defects (43). Moreover, mutations in *SMC1B* result in seminiferous tubules containing only Sertoli cells and spermatogonia in mouse testes, with a complete absence of differentiated spermatocytes or mature sperm. Notably, *COX7A2* is a key gene involved in testosterone synthesis (44). Testosterone, as the principal male sex hormone, binds to androgen receptors on Sertoli cells, indirectly regulating spermatogenic cell development and providing essential nutritional support during spermatogenesis. In this study, transcriptome sequencing was systematically applied to analyze the hypothalamus, pituitary, and testes of Landes geese with high- and low- sperm motility, identifying multiple key genes and signaling pathways closely associated with sperm activity. These findings provide critical theoretical insights into the molecular regulation of male reproductive capacity. Future studies integrating multi omics approaches, such as lipidomics, could systematically characterize the dynamic metabolic features of sperm membrane fatty acids and their intrinsic relationships with spermatogenesis, sperm maturation, and fertilization, thereby offering a theoretical basis for elucidating the molecular mechanisms of sperm function and for genetic improvement of reproductive performance in poultry. The upregulation of these spermatogenesis-related genes in HSM geese further highlights their potential role as candidate molecular markers associated with semen quality. These genes may influence sperm structure, energy metabolism, and meiotic progression, collectively contributing to improved sperm motility.

## 5 Conclusion

In summary, high-sperm motility Landes geese exhibit enhanced antioxidant capacity and endocrine regulation, along with upregulation of genes involved in spermatogenesis and steroidogenesis. Differential gene expression within the hypothalamic–pituitary-testicular axis appears to contribute to variations in sperm motility and overall reproductive performance. These findings provide valuable insights into the molecular mechanisms underlying male reproductive capacity in Landes geese and offer a theoretical basis for improving breeding efficiency.

## Data Availability

The original contributions presented in the study are publicly available. This data can be found here: The data presented in the study are deposited in NCBI SRA database. The accession number is: PRJNA1451947.
