# Supplementary material for: Integrative analysis of the hypothalamic-pituitary-testicular axis reveals molecular mechanisms underlying sperm motility differences in Landes ganders
Source: Front Vet Sci. 2026 Apr 22;13:1809258. doi: 10.3389/fvets.2026.1809258 (PMC13143575; doi:10.3389/fvets.2026.1809258)
Supplement: Supplementary file 1 [file Table_1.docx]

Supplementary Table 1. Primer sequences used for qRT-PCR validation and RNA-Seq related applications.

| **Genes** | **Forward sequence (5'-3')** | **Reverse Sequence (5'-3')** | **NCBI ID** |
| --- | --- | --- | --- |
| *GAPDH* | TGGTGCAAGAGGCATTGCTGAC | GCTGATGCTCCCATGTTCGTGAT | XM_067004670.1 |
| *SPATA1* | TAGAAAGCCCATCTGGCACTC | AGCTCCACCAGCTGGGAT | XM_048059078.2 |
| *3β-HSD* | GACCTGGGGTTTGGAATTGAG | TAGGAGAAGGTGAATGGGGTGT | XM_013196414.3 |
| *CYP11A1* | AGGGAGAAGTTGGGTGTCTACGA | CGTAGGGCTTGTTGCGGTAGT | XM_048081523.2 |
| *CYP19A1* | TGATTGCTGCTCCTGATA | GAGAATAATGTTTGTTCCCT | NM_001347255.2 |
| *STAR* | ACGAATGCAAAACGCCTCG | CCGCTGAAGGATGCTGCG | XM_013194444.3 |
| *SOX9* | TGAAGAGACCCATGAACGCC | CTCTCGTTCAGCAGCCTCC | XM_048064463.2 |
| *WT1* | TACCGGGCTGAGCTTAGGTAG | CAGCAGAGCTCTTTAGAGGCAT | XM_048046719.2 |
| *DNAH7* | TGCAAAATCAGTCCGAGCCT | AGACTCACCTTTTGGCCACT | XM_013187507.3 |
| *SMC1B* | GGCGAGGCTGGGAAAATCT | GGTTTTCCAACATGTGCTCCA | XM_066999992.1 |
| *RPS24* | AACTCACTTTGGTGGTGGCA | CAGCACCGACGTTAGCCTTA | XM_067000290.1 |
| *RPS24* | AACTCACTTTGGTGGTGGCA | CAGCACCGACGTTAGCCTTA | XM_067000290.1 |
| *COX7A2* | GAGGATAATGGCCTCCCAGTG | TCTTGGGCATTGAAGCTACC | XM_066994662.1 |
| *RPS2* | TGCAGAAACAGACTCGTGCT | CCCAATAGCCTCGTCGTACC | XM_013174975.3 |
| *DUSP1* | TCCAGTTTGAGTCGCAGGTC | CCTCTGCTCGTGACAGTGTT | XM_067005574.1 |
| *CXCR4* | ATTCCAGCTAACACTGCCCC | AACCACTTGTCCACAGGACC | XM_048062740.2 |
| *FN1* | CAACGTCAACTGCCCCATTG | AATGGCTCCAGAACGAAGGG | XM_013178607.3 |
