# Supplementary material for: Integrative analysis of the hypothalamic-pituitary-testicular axis reveals molecular mechanisms underlying sperm motility differences in Landes ganders
Source: Front Vet Sci. 2026 Apr 22;13:1809258. doi: 10.3389/fvets.2026.1809258 (PMC13143575; doi:10.3389/fvets.2026.1809258)
Supplement: Supplementary file 2 [file Table_2.docx]

**Supplementary table 2.** Basic information of RNA-seq data

| Sample | Raw reads | Clean reads | Q20 (%) | Q30 (%) | GC content  (%) |
| --- | --- | --- | --- | --- | --- |
| HSMG1 | 46452252 | 44509758 | 98.39 | 95.19 | 49.75 |
| HSMG2 | 46961246 | 45723108 | 98.38 | 95.16 | 48.57 |
| HSMG3 | 46968794 | 45684920 | 98.35 | 95.12 | 49.15 |
| LSMG1 | 46182900 | 44912108 | 98.48 | 95.47 | 48.95 |
| LSMG2 | 42206536 | 40965856 | 98.43 | 95.27 | 47.48 |
| LSMG3 | 49052954 | 47503840 | 98.45 | 95.36 | 49.26 |
| HSMC1 | 46084256 | 44039254 | 98.3 | 95.16 | 50.68 |
| HSMC2 | 46521948 | 44413888 | 98.24 | 94.91 | 50.31 |
| HSMC3 | 47662894 | 46052792 | 98.32 | 95.18 | 50.68 |
| LSMC1 | 49525520 | 44894786 | 98.3 | 95.09 | 50.33 |
| LSMC2 | 46035426 | 45730348 | 98.31 | 95.1 | 50.48 |
| LSMC3 | 46346412 | 42735332 | 98.18 | 94.85 | 50.88 |
| HSMX1 | 41877918 | 41559266 | 98.22 | 94.87 | 50.25 |
| HSMX2 | 48342778 | 45455804 | 98.33 | 95.18 | 49.55 |
| HSMX3 | 51141914 | 50844684 | 98.29 | 95.07 | 50.88 |
| LSMX1 | 46928720 | 46680178 | 98.29 | 95.07 | 50.02 |
| LSMX2 | 45823518 | 45549614 | 98.27 | 95.03 | 49.65 |
| LSMX3 | 47687580 | 45300632 | 98.18 | 94.7 | 50.14 |
