# Supplementary figures and images for: Integrative analysis of the hypothalamic-pituitary-testicular axis reveals molecular mechanisms underlying sperm motility differences in Landes ganders
Source: Front Vet Sci. 2026 Apr 22;13:1809258. doi: 10.3389/fvets.2026.1809258 (PMC13143575; doi:10.3389/fvets.2026.1809258)

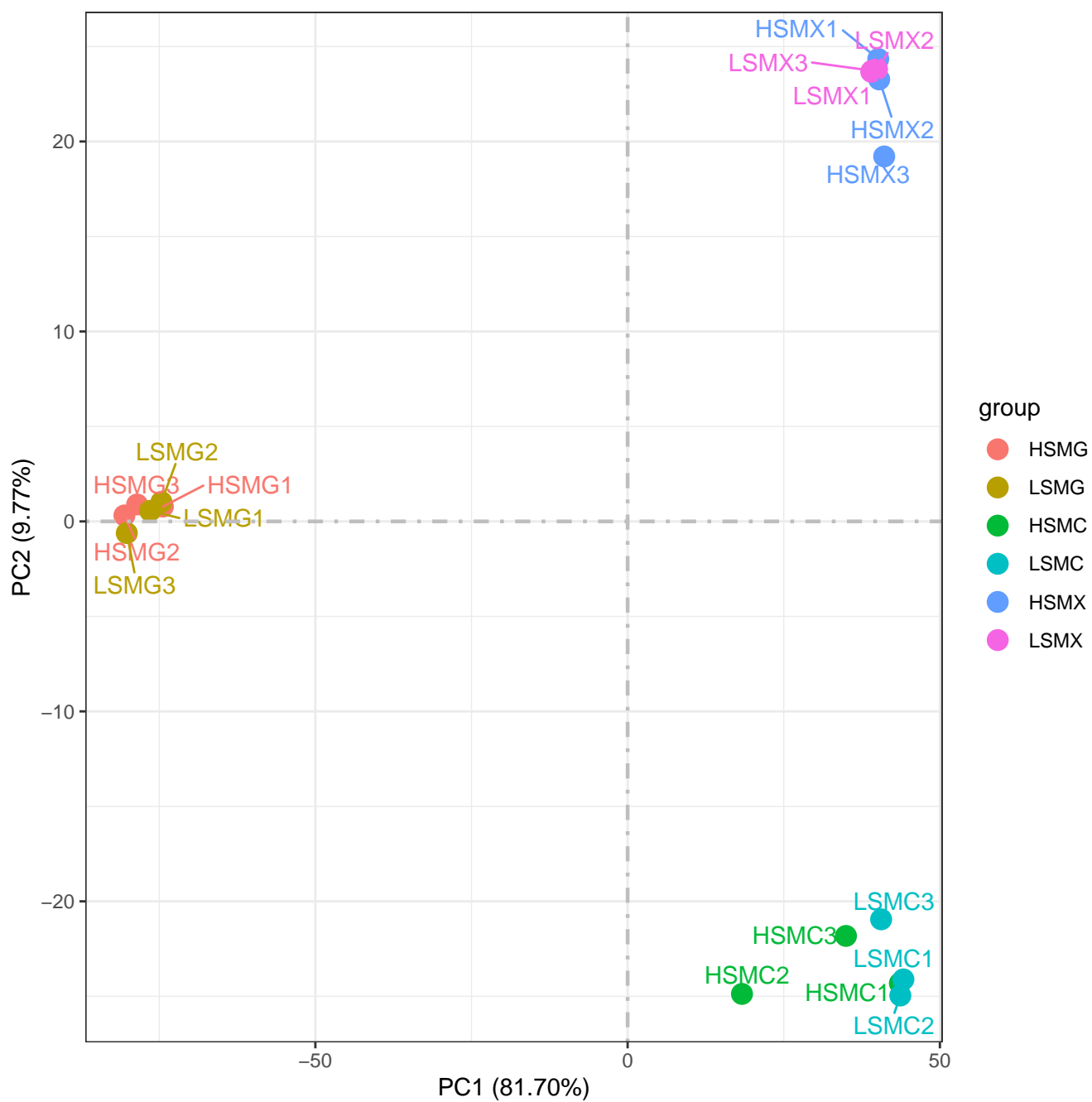

Supplement: Supplementary file 3 [file Image_1.pdf]
